# Comparative effectiveness of an adult social physical play versus traditional group exercise program for adherence and fitness: Protocol for a randomized-controlled trial

**DOI:** 10.1016/j.conctc.2021.100736

**Published:** 2021-02-06

**Authors:** Matthew A. Ladwig, Christopher N. Sciamanna, Liza S. Rovniak, David E. Conroy, Jinger S. Gottschall, Matthew L. Silvis, Joshua M. Smyth, Ming Wang, Brandon J. Auer

**Affiliations:** a Penn State College of Medicine, Hershey, PA, USA; b Penn State University, State College, PA, USA; c Les Mills International, New York, NY

**Keywords:** Physical activity, Promotion, Exercise prescription, Adults, Motivation, Enjoyment, Play, Clinical trials

## Abstract

Despite the myriad benefits associated with regular physical activity (PA), few American adults accrue sufficient weekly PA. Although “lack of time” is often cited as a correlate of physical inactivity, a growing body of evidence suggests that, perhaps more importantly, people allocate their leisure-time to activities they find more enjoyable than PA. These findings underscore the need to devise physical activities that will be chosen over other enjoyable, but less healthy, behavioral alternatives. As a first step in this direction, we designed a group social PA play program for adults, known as PlayFit. The overarching philosophy of PlayFit is that fun and enjoyment are among the most important influences on PA adherence. In PlayFit, traditional sport games are modified to fulfill basic psychological needs, in a non-competitive, and non-contact environment. We will randomize 280 sedentary adults 18–50 years of age to 12-months of PlayFit or traditional group exercise, matching the groups on intensity, frequency, and duration. The primary outcomes include cardiorespiratory fitness (VO_2peak_), group adherence, and group enjoyment. We hypothesize that, at 6 months, cardiorespiratory fitness will have increased to the same extent in both groups, but at 12 months, only those randomized to PlayFit will have maintained their fitness through better adherence than Group Exercise – and this outcome will be mediated by enjoyment of the assigned group. Findings from this study could provide evidence that a focus on providing fun and enjoyable PA experiences for adults may be a viable route toward improving PA adherence. A simple, inexpensive PA intervention, such as PlayFit, may represent one such approach to do so.

## 1 Introduction

Fewer than 1-in-10 American adults meet weekly physical activity (PA) recommendations [1], greatly increasing their chances of developing cardiovascular disease, stroke, and other chronic medical conditions [2]. Despite enormous sums of money and intellectual energy poured into the issue of physical inactivity over the past three decades, little progress has been made in increasing population-level PA. Though adults often cite a variety of barriers to PA (e.g., lack of time, access [3]), a key and possibly modifiable barrier may be that many adults do not enjoy PA enough to participate regularly. For example, monthly gym memberships are used, on average, only once per week, despite the significant costs associated with maintaining membership, and this may be due in part to some individuals not enjoying traditional resistance training and/or machine-based exercises (e.g., treadmill, stationary bicycle) [4].

Given the growing body of evidence suggesting that people who perceive PA as pleasant and enjoyable are more likely to both adopt and adhere to physically active lifestyles [[5], [6], [7], [8], [9], [10], [11], [12], [13], [14], [15], [16]], some authors [7] recommend that PA “interventions should perhaps *initially focus* [emphases added] on increasing enjoyment of physical activity” followed by other known barriers. Furthermore, others [17,18] have suggested that traditional exercise prescriptions (i.e., those that focus primarily on participant safety and efficacy) could be rendered more effective by including an additional overarching consideration; specifically, whether the individual perceives the prescribed exercise to be pleasant and enjoyable. Williams and Evans proposed that repeated experiences of enjoyment during PA might perpetuate subsequent PA by leading to positive affective forecasts (i.e., whether the individual expects to feel good or bad) of subsequent PA [19]. Indeed, unless feelings related to the thought of being physically active are associated with pleasure and enjoyment, discovering the motivation to will oneself to engage in PA may be difficult in the midst of pleasant and enjoyable, but sedentary, alternatives (e.g., using a smartphone, reading or watching television) [[20], [21], [22], [23]].

Although several studies among younger children have attempted to increase exercise enjoyment [24], most of the extant work among adults has measured enjoyment responses to existing exercise regimens (e.g., high-intensity interval training versus moderate-intensity continuous exercise [25]), as opposed to directly comparing an established program (e.g., group exercise) to one matched on intensity, frequency, and duration but designed with the overarching philosophy of maximizing participant enjoyment. Moreover, few studies have investigated whether this primary focus on PA enjoyment would lead to better adherence, and, consequently, impart more health and fitness benefits. With this gap in mind, we began work to design a group PA program, known as PlayFit, specifically engineered to maximize enjoyment (see Table 1 for comparisons between PlayFit and recreational sport leagues) [26]. In PlayFit, we leverage several factors that are theorized to influence PA enjoyment, such as aspects of self-determination theory [27] and hedonic theory (i.e., pleasant experiences are more likely to be repeated [28]). The games of PlayFit are modified to make the games easier to play (i.e., to increase feelings of competence), allow for self-pacing and self-regulation of exercise intensity (i.e., to foster autonomy), and cultivate an environment conducive to positive social interactions (i.e., to improve feelings of relatedness). We hypothesize that the qualities of PlayFit could increase feelings of pleasure and perceived enjoyment of PA leading to a cycle of positive experiences that encourage PA adherence.

**Table 1:** Features of PlayFit versus traditional adult sport leagues.

	PlayFit	Sport Leagues
Keeping score	No	Yes
Tracking standings	No	Yes
Static team membership	No	Yes
Single-sport	No	Yes
Player contact allowed	No	Yes
Official sport rules	No	Yes

## 2 Methods

### 2.1 Overview

The currently proposed study is a community-based, pragmatic randomized-controlled trial. The aims of this study are to 1) test the impact of PlayFit, compared to Small Team Training (STT; i.e., group exercise), on cardiorespiratory fitness (i.e., VO_2peak_) and moderate-to vigorous-intensity physical activity (MVPA; self-reported and objectively monitored) after 6-and 12-months, and 2) examine group differences in rates of injury and PA program enjoyment and changes in self-efficacy, blood pressure, loneliness, anxiety, and depression. We hypothesize that participants randomized to both groups will improve their cardiorespiratory fitness similarly at month-6 (i.e., approximately 3.5 ml/kg/min), but those randomized to PlayFit will maintain these increases at month-12 through better attendance. Additionally, we hypothesize that changes in group attendance and cardiorespiratory fitness at month-12 will be mediated by group enjoyment. Finally, we hypothesize that participants randomized to PlayFit will demonstrate greater improvements in indices of physical health (i.e., blood pressure) and mental health-related (i.e., anxiety) outcomes compared to STT while reporting similar injury rates. All study procedures presented here have been approved by the Institutional Review Board (IRB).

### 2.2 Participants

#### 2.2.1 Eligibility criteria

Participants will be healthy, but sedentary, adults between 18 and 50 years of age [29]. The participatns will be considered “sedentary” if they report fewer than 90-minutes of MVPA each week [30], based on questions provided in the International Physical Activity Questionnaire (IPAQ; [31]). Finally, because some participants who are screened will be at increased risk for heart disease (and others may have unrecognized symptoms [32]), a physician must provide clearance for participants who answer affirmatively to any item on the Physical Activity Readiness Questionnaire (PAR-Q; [33]). This clearance is particularly important as the proposed physical activities will be moderate-to vigorous-intensity [34].

#### 2.2.2 Recruitment and screening

We will send recruitment letters to patients in the Penn State Health system who reside within a 10-mile radius of each exercise site. If our recruitment rate is unsatisfactory, we will also use a commercial mailing list provider, such as Lorton Data (Arden Hills, MN). To ensure eligibility and review the study procedures, trained research staff will screen individuals over the phone using a standardized script. Prior to enrollment and any study measures, eligible participants will attend a 60-minute orientation session led by trained research staff in a designated location near each exercise site. Similar orientation sessions have successfully improved retention in weight-loss trials by exposing potential participants to more information about the study and its expectations. The orientation session will include: 1) addressing the time and scheduling demands of enrolling and participating in a 12-month RCT (e.g., considering realizing the difficulty associated with travel to PA program sites/measurement sessions), 2) the barriers to PA change after having been chronically sedentary, overweight, and/or obese (e.g., soreness), and 3) managing expectations for individuals and groups (e.g., not being randomized to the group he or she was hoping to be) [35]. In addition, because commitment devices have been shown to positively influence habit formation [36], at the end of the orientation session, we will ask participants still interested in enrolling to make an informal commitment to attend at least 2 out of 4 exercise sessions per week, by viewing the schedule of available exercise sessions, and personally initialing the days that they intend to participate on a monthly basis. If the individual remains interested after the orientation session, he or she will schedule a baseline visit to sign the informed consent and collect initial measurements.

### 2.3 Measures

#### 2.3.1 Primary outcomes

**Cardiorespiratory fitness (VO**_**2peak**_**).** Cardiorespiratory fitness at baseline and month-6 and-12 will be assessed using a graded-exercise test (GXT). The GXT will be performed using a Bruce protocol [37] on a computerized treadmill while breath-by-breath data is collected using a two-way valve attached to a metabolic measurement system, such as Parvomedics (Salt Lake City, UT). Prior to each test, the system will be calibrated for oxygen and carbon dioxide using a certified mixture of the gases and for ventilation using a 3-l syringe and a standard 15-stroke calibration procedure. During each GXT, a clinical exercise physiologist will collect heart rate and blood pressure measurements, as well as monitor electrocardiography (ECG) data for adverse responses. The test will be terminated at the point of volitional exhaustion. We will consider VO_2peak_ as the point at which three or more of the following criteria are met: 1) a plateau in VO_2_ despite increasing running speed, 2) a respiratory exchange ratio (RER) higher than 1.1, 3) an inability to maintain the required velocity, or 4) heart rate above 90% of age-predicted maximum [38].

**Weekly moderate-to vigorous-intensity physical activity.** We will use tri-axial accelerometers (ActiGraph GT3X, Pensacola, FL) to measure ambulatory MVPA. The accelerometer will be worn for 7-days following each in-person exercise test visit (baseline, 6, 12-month). Research staff will ensure that participants understand how to correctly wear the accelerometer and will send daily reminders to wear the device during the accelerometer wear period. These data will be stored as 10-second epochs, based on regression equations determined by Crouter and colleagues [39], who tested a range of sports and exercises characterized by intermittent bursts of activity similar to the sports and exercises in PlayFit and STT. We will derive minutes-per-day that each participant spends in MVPA (>2,019 counts per minute) using the cut-points recommended by Matthew and colleagues [40]. Researchers using the ActiGraph and similar tri-axial accelerometers have reported excellent reliability among other adult samples [41,42]. We will inspect these data for quality assurance by requiring participants to provide at least 10-hours of waking wear time each day, use paper-and-pencil activity logs to determine non-wear periods, and require at least 5 valid days or 66 valid hours over 7-days and at least one weekend day of wearing the device [43].

### 2.4 Secondary dependent measures

#### 2.4.1 Mediators

All questionnaire-based measurements will be collected electronically using the Research Electronic Data Capture (REDCap [44]) system, a secure, web-based software platform.

**Physical activity enjoyment.** Participants will rate their perceived enjoyment of PA at baseline, 6, and 12-months using the Physical Activity Enjoyment Scale (PACES [45]), an 18-item questionnaire with 7-point bipolar scales where a higher score indicates greater perceived enjoyment. The stem, “Think about the exercise you have been doing…” is followed by items such as, “something I liked–something I disliked”. We will modify the stem to target specific enjoyment for the group to which the participant is assigned. (e.g., “Think about the exercise group you have been participating in during this study…"). The PACES has demonstrated excellent reliability in other adult samples [45].

**Exercise adherence.** Daily attendance logs will be maintained by the exercise leaders supervising each group.

**Self-reported physical activity.** Participants will self-report PA using the long-form of the International Physical Activity Questionnaire (IPAQ; [31]). The 31-item IPAQ was designed to provide an evaluation of PA in four domains: work, household, transportation, and leisure. A reference period of “during the last 7 days” will be used when collecting responses. The eight-day test-retest reliability for PA recall of the IPAQ was good with a median coefficient of *r* = 0.80. Criterion validity using accelerometers was acceptable with a median coefficient of 0.40 [46].

**Adverse events and injuries.** We will assess injuries and other adverse events monthly, using a self-report measure adapted from the one developed by Stathokostas and colleagues [47]. The questionnaire consists of 7-items that focus on the region of the body that is injured as well as the injury severity (i.e., whether the participant should contact or visit his or her PCP). Stathokostas and colleagues reported high test–retest reliability (*r* = 0.76) for this measure, with interrater agreement coefficients (*κ*) greater than 0.80.

**Depression and anxiety.** We will use 11 self-reported questions derived from the National Institutes of Health (NIH)-supported Patient Reported Outcomes Measurement Information System (PROMIS) to assess depression, anxiety, and loneliness. Choi and colleagues reported excellent reliability (*r* = 0.91–0.98) for these measures [48]. Scores from theses subscales are positively correlated with valid other “gold standard” measures [49,50], and are responsive to treatment effects [51]. Participants scoring one or more standard deviations above the mean on the depression and/or anxiety subscales will be contacted by research staff within 48 hours and encouraged to speak with their PCP about their results.

**Loneliness.** The Lubben Social Network Scale (LSNS6) will be used to measure participant loneliness. The LSNS6 consists of 6-items intended to measure the extent of a person's social engagement with both family (3 items) and friends (3 items) [52]. Scores from the LSNS6 exhibit good test-retest reliability (*r* = 0.83) and are associated with future mental health outcomes, such as depression and dementia [53,54].

**Body weight and height.** Body weight and height will be measured using a calibrated scale and stadiometer (Tanita, Inc) [55] during the exercise test visits.

**Blood pressure.** Blood pressure will be assessed during the exercise test visits according to the recommendations of the American Heart Association [56]. Resting blood pressure will be measured with the participant in a seated position using a calibrated aneroid sphygmomanometer.

### 2.5 Covariates and other variables

PA **program satisfaction.** Satisfaction for the assigned group (i.e., net promoter score) will be measured at the end of each month using the question: “How likely are you to recommend this program to a friend?” using a 10-point (0 = Not at all likely, 10 = Extremely likely) scale. This single-item question has shown to be predictive of future sales growth in marketing studies [57]. In addition, we developed several face-valid questions to measure anticipatory enjoyment, including 1) “How much, if at all, do you look forward to the exercise sessions?” (0 = Not at all, 4 = Very much), 2) “Have you gotten together, outside of these exercise classes, with someone you met in this program?”, and three open-ended items including 1) "What's the one thing we could do to improve the exercise program?”, 2) "What's the one thing you like best about the exercise program?”, 3) “In one sentence, how would you describe what these exercise sessions are like, if a friend asked you.” A final question will also be posed to the participant to rate the exercise leader who led their most recently attended session, “How would you rate this instructor?” (0 = Poor, 4 = Excellent).

**Exercise self-efficacy.** Exercise self-efficacy will be measured using the 5-item self-efficacy scale developed by Marcus and colleagues [58]. Other studies have reported good internal consistency (Cronbach's *α* = 0.76) and test-retest reliability over a 2-week period (*r =* 0.90) [7].

**Preference for exercising in groups versus alone.** This 4-item measure has been used to assess the preference of adults for exercising alone versus in groups, and in different age groups [59], on a 5-point Likert-type scale ranging from −2 (Very unappealing) to + 2 (Very appealing).

**Competitiveness.** We will assess competitiveness using a 12-item measure developed by Conroy and colleagues [60] that assesses the degree to which subjects endorse varying achievement goals during sport. The Achievement Goal Questionnaire for Sport (AGQ-S) measures four goals: Mastery approach (MAp), Mastery avoidance (MAv), Performance approach (PAp), Performance avoidance (PAv), with 3-items for each construct. Overall, the AGQ-S scores have demonstrated strong psychometric properties with evidence of good 7-day differential stability (and test-retest reliability): MAp 0.77 (0.65), MAv 0.60 (0.55), PAp 0.87 (0.76), PAv 0.80 (0.73), latent mean stability, longitudinal factorial invariance, and external validity [60].

**Sociodemographics and tobacco use.** We will measure age, gender, race and ethnicity, smoking status and education, using standard items [[61], [62], [63]].

## 3 Procedure

### 3.1 Blinding

The research staff that deliver the intervention will be blinded to the research hypotheses, and those who administer the exercise testing will be blind to both the research hypotheses and the group assignment of each participant.

### 3.2 Randomization

**Fig. 1:**
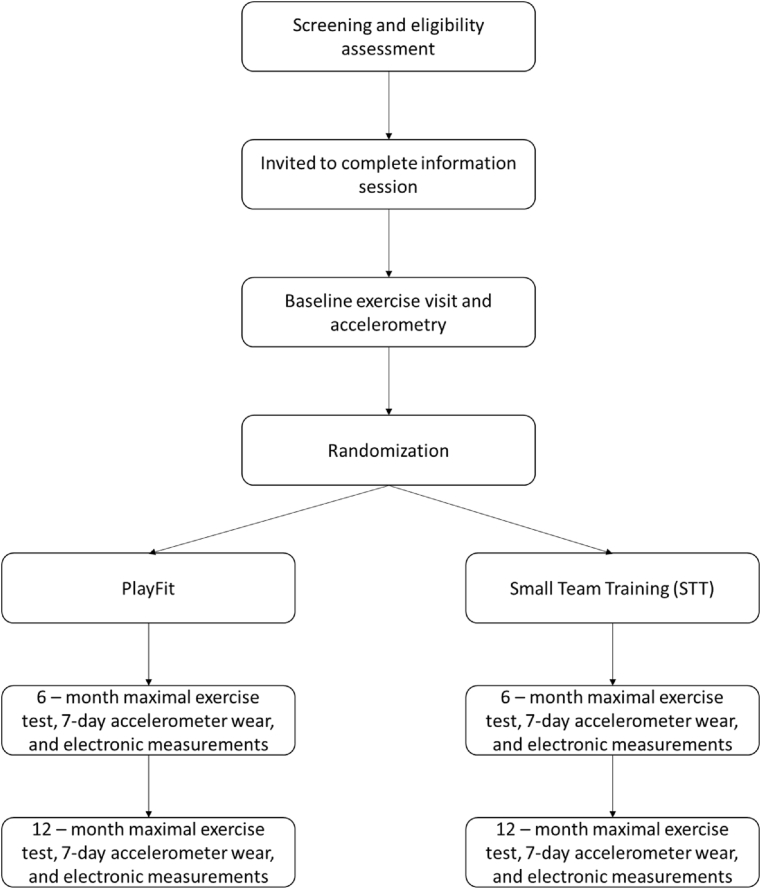
Study flow-chart.

Following the baseline exercise visit and accelerometer wear period (see Fig. 1), participants will be radnomly assigned using stratified assignment by age (i.e., those over and under 35 years of age) and biological sex. The randomization will be performed using REDCap software. Following randomization, a research staff member will contact the participants to review their exercise group assignment, where the sessions will be held, what they should bring to each session, and answer any additional questions. Additionally, participants will be given the option to install a smartphone application to receive session confirmations and, if necessary, cancellation notifications from the PA program leaders.

Participants will be invited to attend up to 4 sessions per week of their assigned group. Similar to the PA programs offered at many health clubs, we will provide different session time options during the week to overcome scheduling barriers. The leaders of both PA programs will be trained to deliver the interventions by the investigators (MAL, CNS, JSG) and evaluated regularly to ensure fidelity.

#### 3.2.1 PlayFit

Each 1-hour PlayFit session will begin with a 5-minute dynamic warmup period of “catch-and-chat”, with all group members, using the sport implement of the day (e.g., soccer ball, Frisbee). Following the warmup, participants will complete up-to 8-minute play periods (i.e., 4mins, 6mins, 8mins per play period during weeks 0–2, 3–5, and 6-weeks to 12-months, respectively, to allow for gradual increase in PA volume) with each play period followed by a 5-minute rest/water break. The warmup and rest breaks are designed to encourage socialization. The exercise leader who oversees the sessions will provide a basic set of rules for each sport during the warmup (see Table 2 for rules for each sport game). The five sport games of PlayFit include soccer and 4 others that each use the rules of ultimate Frisbee (i.e., handball, netball, Ultimate football). Each week, the games played will be rotated, to provide variety. The instructor will also play while supervising to ensure safety and that the core philosophy of PlayFit (i.e., to maximize enjoyment) is maintained. In addition to considering the tenets of self-determination theory, hedonic theory, and interventions among children, some of the additional approaches we use to maximize enjoyment include that the leaders randomly select teams (i.e, no team captains) and switch after each play period, players may rotate in and out for breaks if and whenever they please (i.e., teams do not need to be even at all times), de-emphasizing inter-individual competition (e.g., we do not keep score), positive, generalized encouragement (e.g., no trash-talking, leaders do not criticize performances), and no-contact (i.e., maintain arms-length distance from one another while playing, do not kick at the feet of others). Following the final play period, another 5-minute period of “catch” will allow for a cooldown and more socializing.

**Table 2:** General and specific rules and equipment used during PlayFit games.

General Rules		
	• No penalties, players may position themselves anywhere. • Maintain arms-length distance from others when playing • Play begins at midfield/court. • Each period, alternate who starts with possession. • Possession goes to other team after a goal/touchdown. • If ball goes “out of bounds”, an opposing player returns ball to play.	
Game	Equipment	Additional Specific Rules
Soccer	Field markers (cones) Lightweight volleyball, inflated to 2.0 pounds per square inch (PSI). 4′ × 6′ collapsible goal.	• No goaltenders • No digging (kicking) at the feet of others
Ultimate Frisbee™	Field markers (cones) Soft-sided, flexible Frisbee™	• Players may take up to 2-steps before passing to teammate or attempting to score. • Players can hold the ball for up to 3-seconds before needing to pass or attempt to score.
Ultimate football	Field markers (cones) Nerf™ foam football	• Players may take up to 2-steps before passing to teammate or attempting to score. • Players may hold ball for up to 3-seconds before needing to pass or attempt to score.
Handball	Field markers (cones) Voit™ Tuff Coated Handball (6 inches, 5.8 oz) 4′ × 6′ collapsible goal.	• No goaltenders • Players may take up to 2-steps before passing to teammate or attempting to score. • Players may hold ball for up to 3-seconds before needing to pass or attempt to score.
Netball	Field markers (cones) 2 regulation-height basketball hoops. Standard adult-sized basketball inflated to 8.0 PSI	• Players may take up to 2-steps before passing to teammate or attempting to score. • Players may hold ball for up to 3-seconds before needing to pass or attempt to score. • Players may attempt baskets from any location on the playing area.

#### 3.2.2 Small Team Training

The STT condition is designed to represent a “standard-of-care” for PA programming, as instructor-led group exercise classes are common at most fitness centers. We have selected a set of movements from common fitness regimens to match the energy expenditure of PlayFit. STT consists of a variety of bodyweight interval exercises. The sessions will consist of: 5 minutes of greetings, small talk, question of the day, 5 minutes of easy movement, 5 minutes of explanation of training session, demonstration of exercises, practice novel or complex moves, followed by 20-30 minutes of training session, and the final five minutes are devoted to stretching and praise. These exercise offered in the sessions will change weekly and will be included in a week-by-week plan for leaders of the Group Exercise condition. In addition, participants will be encouraged to ask questions of the Group Exercise leader at the beginning and/or end of the classes, as this is the standard of care at fitness centers.Because group fitness classes are usually attended by more women than men, we will design the intervention to be attractive to both sexes, by incorporating components of strength training, athletic movements (e.g., high knee runs, block jumps), and martial arts (e.g., kicks, jabs).

## 4 Data analysis

### 4.1 Sample size determination

Our power and sample size calculations are based on the following considerations. First, studies among untrained adults have shown that supervised group exercise can increase VO_2peak_ by approximately 4 ml/kg/min over 12 months [[64], [65], [66]]. Based on data derived from the National Health and Nutrition Examination Survey (NHANES) among men (*mean*_VO2peak_ = 42.1 ml/kg/minute) and women (*mean*_VO2peak_ = 34.2 ml/kg/minute) 30–39 years of age [67], we assume the baseline VO_2peak_ of participants will be approximately 35.0 (SD = 6.0). The SD of 6.0 is a conservative estimate using data from the Naval Research Lab, where it was 6.3 and 3.9 in men and women, respectively [68]. After 6-months, we anticipate that participants in both PlayFit and STT will improve their VO_2peak_ by approximately 10% (i.e., 3.5 ml/kg/min), owing to similar rates of program adherence. However, after 12-months, we anticipate only those randomized to PlayFit will maintain these changes in cardiorespiratory fitness, due to better program adherence. These differences in adherence will lead to a maintained improvement (versus baseline) in VO_2peak_ of PlayFit participants at 12-months of 3.5 ± 6.0 ml/kg/minute versus 1.0 ± 6.0 for STT. In this case, sample sizes of 112 in each group achieve 80% power to detect a mean difference of 2.5 ml/kg/minute with a standard deviation of 6.0 at the first time point, a standard deviation of 6.0 at the second time point, a two-sided significance level (*α*) of 0.05, and a correlation between measurements of 0.39. To account for participant attrition, we will oversample at a rate of 25%. Therefore, we will recruit up to 280 participants for the proposed study.

#### 4.1.1 Preliminary data analysis plan

Given the relationship between cardiorespiratory fitness and mortality [69], we will conduct the primary analysis on changes in fitness. First, to ensure successful randomization, the two groups will be examined for homogeneity. If it is determined that the groups differ significantly on any variable, we will include those variables as covariates in subsequent analyses. For the primary outcome (VO_2peak_), we will apply intention-to-treat (ITT) principles with all available data included in the first set of analyses [70,71], with the assumption that participants who are lost to follow-up will not have changed their level of fitness since their last observation.

#### 4.1.2 Missing data

If the combined rate of missingness on a variable is less than 5%, we will not impute missing data. In the event of unanticipated higher levels of missing data, we will examine the nature of the missing values. If it is believed that variations in attrition can be explained by observed variables, these data will be considered missing at random (MAR) and no special adjustments will be necessary [70]. If, instead, we find that a portion of the variability in attrition could be explained further by unobserved variables, we will employ multiple imputation, carrying the last observation forward.

#### 4.1.3 Analysis of main outcome: fitness changes

To conduct these analyses using the principles of ITT, a slope will be fitted for the outcome variable (i.e., VO_2peak_) across each time point. Provided statistical assumptions are met, our hypotheses will be tested using random regression models [72,73]. Random regression models, also known as growth models, help to attenuate the impact of random data fluctuations, thereby increasing statistical power [72,74]. The use of random regression models also accounts for data non-independence, missing data, and nonlinear changes in the outcome variables [75].

#### 4.1.4 Analyses of secondary outcomes

We will analyze the effect of each condition on each physical health and mental health-related outcome. Hockberg's step-down procedure will be used to provide corrected and uncorrected *p*-values for the entire set of secondary outcomes [76]. To conduct these analyses by ITT, a slope will be fitted for each outcome variable across all time points. As before, these hypotheses will be tested using random regression models.

#### 4.1.5 Mediator analysis

We propose two potential mediators of fitness in the current study, perceived enjoyment and session attendance for each exercise group. We will examine these potential mediators using the analytical approach suggested by Baron and Kenny [77] and others [78]. Multiple regression analyses will be conducted to obtain the direct effects of the relationship between group assignment and fitness followed by examining the indirect effects of adding perceived enjoyment and attendance to the respective models.

## 5 Discussion

Because perceptions of pleasure and enjoyment during PA have increasingly been found to be associated with adherence [[5], [6], [7], [8], [9], [10],^17^], it begs the question of "if adults were offered access to an PA program specifically designed to maximize enjoyment, would their adherence and, subsequently, health and fitness outcomes be better than those of traditional group exercise programs?" Most contemporary models of PA promotion focus on improving adherence via goal-setting, tracking progress, providing feedback, and overcoming barriers [17]. The questions we aim to answer in this proposed study are complementary to those lines of work, but expand the range of potential influences to include the design and composition of the PA program itself. We hypothesize that, by offering repeated experiences of enjoyable PA, participants randomly assigned to the PlayFit condition will form associations between PlayFit and enjoyment, thus improving their likelihood of adherence [[19], [20], [21]].

Because most fitness centers offer group exercise classes, and to control for the effect of exercising alone versus in groups, we have selected group exercise as our comparator. In most group exercise classes, the leaders provide the only direct interaction with participants, in the form of instruction. Beyond social interaction being optional in most group exercise classes, the activities are also usually engineered primarily to be to be safe and effective (e.g., lead to changes fitness, weight, and/or in body composition). In contrast, the overriding philosophy of PlayFit is to first and foremost, maximize enjoyment, by promoting relatedness through positive social interactions using team-based gameplay a, improving perceptions of competence by making the games easier to play, and increasing autonomy through encouraging self-pacing and regulation of PA intensity. If shown to be effective, the design approach of PlayFit could be used to provide more enjoyable alternatives for adults who may prefer playful, laid-back, non-competitive exercise options, versus participation in often-competitive “adult” or recreational leagues [79] or group exercise classes. Despite the importance of PA play to the human experience and its ubiquity among normally-developing children, few adults continue to play sports [80] and little work has attempted to apply aspects of PA play to adult PA promotion [11] and approaches like PlayFit may help remedy many of the same factors suggested to drive attrition from youth sport [81,82].

If PlayFit outperforms STT, we believe that several things may occur. First, fitness centers could offer programs like PlayFit as alternatives to traditional recreational leagues and group exercise classes for adults that maximize positive social interactions. This approach could help members form new relationships which could, in turn, help clients maintain their memberships [83]. Another positive aspect of PlayFit is that because it can implemented with low costs and little setup/equipment on a basketball court or other similarly-sized indoor or outdoor space, many fitness centers and parks will already have the necessary infrastructure in place. The design philosophy of PlayFit may also lay the foundation for changes to the advice fitness and medical professionals provide to their clients. Rather than focusing primarily on criteria such as the duration and intensity of PA, providers could encourage patients to seek out those activities that they find most enjoyable (i.e., a tripartite versus bipartite approach to exercise prescription [17,18]). A focus on first fostering enjoyment during PA promotion may help set the stage for the improvements in PA adoption and adherence long sought-after by exercise scientists..

## Declaration of competing interest

Christopher Sciamanna has an investment, such as stock, in a company which has begun to investigate the possibility of creating a business that provides exercise programs. There are no further known conflicts of interest to declare.
